# Complete Coding Genome Sequence of a Putative Novel Teschovirus Serotype 12 Strain

**DOI:** 10.1128/genomeA.00107-16

**Published:** 2016-03-10

**Authors:** C. Cano-Gómez, M. A. Jiménez-Clavero

**Affiliations:** Centro de Investigación en Sanidad Animal (CISA), INIA, Valdeolmos, Spain

## Abstract

Porcine teschoviruses are ubiquitous and prevalent viruses generally harmless to their hosts, the suids. Here, we report the first complete coding genome sequence of a putative new serotype of porcine teschovirus (PTV-12), strain CC25, isolated from fecal material from a healthy pig in Spain.

## GENOME ANNOUNCEMENT

Porcine teschoviruses (PTV) (*Teschovirus* genus, family *Picornaviridae*) comprise 11 known serotypes to date (PTV-1 to PTV-11) (1). Specific to swine, most PTVs are usually nonpathogenic, but some viral variants cause severe (Teschen disease) or mild (Talfan disease) disorders in the central nervous system, as well as reproductive, digestive, and respiratory distresses (2). PTV are positive-sense single-stranded RNA viruses approximately 7.1 kb long, which contain one open reading frame encoding a single polyprotein flanked by 5′- and 3′-nontranslated regions (NTRs) and a poly(A) tail at its 3′ end. Proteolytic cleavage of the polyprotein leads to 3 precursor polypeptides (P1, P2, and P3), which are subsequently cleaved into 12 mature products: a leader protein, four structural proteins (VP1 to VP4, derived from P1), four nonstructural proteins (from P2), a genome-linked protein, a protease, and the polymerase (from P3).

Previous studies revealed a high genetic diversity of PTVs circulating in Spanish healthy pigs (3, 4). Phylogenetic analysis based on the VP1 coding sequence grouped all these isolates into 12 clusters, suggesting the existence of a 12th serotype (4). In the present study, an isolate representative of this new cluster, named CC25, was characterized in depth for the first time, including its complete coding sequence, allowing an evaluation of its relatedness with other PTV serotypes.

CC25 RNA was extracted from 100 µl of virus-infected IB-RS_2_ cell supernatant. Initially, genomic fragments derived from the VP1 and VP2 coding regions were amplified using published primers (4, 5). To complete the full coding genome, 15 primer sets were specifically designed based on the alignment of representative sequences of all known teschovirus serotypes. A multiple alignment of complete P1 sequences, including 74 strains available in GenBank, was carried out by Clustal X. Phylogeny reconstruction was made using the maximum likelihood statistical method, conducted by MEGA 5.05.

A partial genome of 6,952 nucleotides was obtained, including partial 5′ and 3′ NTRs and the complete polyprotein coding region. Identity analyses of the P1 region showed greater homology with genotypes 2, 4, 6, and 8 at both the nucleotide and amino acid levels, ranging from 81 to 82% and 86 to 87% of maximum identity, respectively, while displaying lower nucleotide and amino acid identities (75% and 79%, respectively) to the remaining genotypes (1, 3, 5, 7, and 9–11). Phylogenetic analysis in the structural region (P1) grouped the sequences into 12 distinct clusters, and the tree topology corroborated the monophyly of genotypes 2, 4, 6, 8, and 12 that was previously established (4).

In this study, sequence and homology analyses showed that isolate CC25 represents a new genotype among the *Teschovirus* genus, which may putatively constitute a novel serotype, PTV-12, based on (i) its nucleotide and amino acid sequence identities in the polyprotein P1, which are as low as those existing between the different serotypes, and (ii) the fact that it clusters separately in a P1-based phylogenetic tree.

The PTV-12 sequence provides more information about the genetic diversity of teschoviruses and suggests further studies be conducted regarding the serological relatedness with other PTVs.

### Nucleotide sequence accession number.

The complete coding genome sequence of PTV-12 (strain CC25) was deposited in GenBank under accession no. JN859128.
